# Impact of 2 Versus 1 Colostrum Meals on Failure of Transfer of Passive Immunity, Pre-Weaning Morbidity and Mortality, and Performance of Dairy Calves in a Large Dairy Herd

**DOI:** 10.3390/ani11030782

**Published:** 2021-03-11

**Authors:** Angel Abuelo, Faith Cullens, Amanda Hanes, Jill L. Brester

**Affiliations:** 1 Department of Large Animal Clinical Sciences, College of Veterinary Medicine, Michigan State University, East Lansing, MI 48824, USA; hanesama@msu.edu (A.H.); brester@msu.edu (J.L.B.); 2 Agriculture and Agribusiness Institute, MSU Extension, Michigan State University, East Lansing, MI 48824, USA; cullensf@msu.edu

**Keywords:** bovine respiratory disease, calf health, diarrhea, heifer management, reproduction

## Abstract

**Simple Summary:**

This retrospective study of records from a dairy farm explored the impact of receiving a second feeding of colostrum 5–6 h after an initial meal of 3 L of quality-tested colostrum on calf health and productivity until the first lactation. Calves that received a second feeding were less likely to develop failure of transfer of passive immunity or morbidity pre-weaning than calves only receiving one feeding. Similarly, calves receiving a second feeding showed a greater growth rate until weaning and tended to produce more milk in the first lactation. However, receiving a second feeding was not associated with differences in pre-weaning mortality or reproductive efficiency. Our results show that feeding calves a second meal of colostrum within the first few hours of life has a positive impact on calf health and might have positive effects into the first lactation.

**Abstract:**

Failure of transfer of passive immunity (FTPI) due to inadequate ingestion of colostral immunoglobulins by calves is associated with increased mortality and morbidity risks. Feeding calves a sufficient amount of quality-tested colostrum within the first hours of life is essential for successfully transferring passive immunity. Many farms have implemented a second meal of colostrum to maximize the opportunities for passive immunity transfer. However, excellent passive immunity can be achieved with a single feeding of sufficient quality-tested colostrum. Moreover, there is currently no evidence demonstrating the impact of a second colostrum feeding within 24 h of life in calves receiving adequate volumes of quality-tested colostrum in an initial feeding. Hence, the objective of this retrospective cohort study was to compare the risks of FPTI, pre-weaning morbidity and mortality, and growth and performance between dairy calves that received one or two feedings of colostrum. For this, the health and production records of a large dairy herd were analyzed. At this farm, newborn calves receive 3 L of quality-tested colostrum soon after birth, followed by another 2 L 5–6 h later. However, at times of shortages of colostrum, calves only receive the initial 3 L meal. The records of 2064 male and 2272 female calves were analyzed, where 4156 and 180 calves received 2 and 1 colostrum meals, respectively. Data from both sexes were included in the analysis of the risks of FTPI, morbidity, and mortality; however, only data from heifer calves were utilized for growth and performance analysis. Survival analysis, and logistic and linear regression models were used to investigate the impact of receiving two feedings of colostrum on calf FTPI status, morbidity, mortality, reproductive indices, pre-weaning average daily gain (ADG), and first lactation 305-d Mature Equivalent milk production (305ME). Calves that received two feedings of colostrum had lower odds of FTPI, a lower probability of being treated for respiratory disease, diarrhea, or any disease, and a greater pre-weaning ADG. However, there was no association between the number of colostrum feedings and pre-weaning mortality, and the probabilities of first insemination and first calving, although heifers receiving two colostrum feedings tended to receive fewer inseminations and to have a greater first lactation 305ME. Collectively, our results suggest that feeding calves a second feeding of colostrum 5–6 h after the initial feeding soon after birth could be an effective strategy to decrease FTPI and morbidity and optimize ADG in dairy calves pre-weaning.

## 1. Introduction

Calves are born with a naïve immune system, thus relying on passive immunity transfer via colostrum to help fight infections during the first weeks of life [1,2,3]. Failure of transfer of passive immunity (FTPI) is associated with increased mortality and morbidity risks and long-term decreased performance including milk yield and longevity in lactating cows [4,5,6]. The prevalence of FTPI among dairy heifers continues to be high worldwide, with recent estimates of 13.0% in the US [7], 23.6% in Canada [8], 32.3% in New Zealand [9], or 41.9% in Australia [10]. Each case of FTPI has an average estimated cost of 60 Euro (72.3 USD (EUR = 1.20 USD on 17 February 2021)) [4]. Thus, FTPI is an important concern in the dairy industry, affecting calf health and farm profitability.

Feeding a low total volume of colostrum in the first 24 h of life is a risk factor for FPTI [11]. Thus, it is recommended that calves be fed 10 to 12% of their bodyweight within 1 to 2 h after birth to transfer passive immunity successfully [12]. This has resulted in a decrease in the US national prevalence of FPTI in the last decades, although without a reduction in pre-weaning morbidity and mortality [13]. In addition, a study conducted in 50 Michigan dairies showed that the farms with the lowest FTPI prevalence all fed a second feeding of 2 to 3 L of colostrum 6 to 12 h after the first feeding [14]. Consequently, offering a second colostrum feeding to calves has been implemented by many dairy farms [15] in an attempt to optimize passive immunity transfer.

However, excellent passive immunity (≥25 g/L serum IgG) can be achieved with a single colostrum feeding [13], suggesting that a second meal might not be needed for adequate transfer of immunoglobulins. Colostrum is also rich in other nutrients and bioactive compounds that influence post-natal development, metabolism, and growth [16,17,18]. Therefore, the benefits of a second colostrum meal need to be evaluated. To our knowledge, however, there is currently no evidence demonstrating the impact of a second colostrum feeding within 24 h of life in calves receiving adequate volumes of quality-tested colostrum in the single feeding. Thus, this study’s objective was to compare the risks of FPTI, pre-weaning morbidity and mortality, and growth and performance between dairy calves that received two feedings of colostrum and those that only received one feeding after birth. For this, we conducted a retrospective cohort study analyzing the records of a large dairy herd. We hypothesized that dairy calves receiving a second colostrum feeding would have lower risks of FTPI, pre-weaning morbidity and mortality, and show greater ADG, reproductive efficiency, and first lactation milk yield.

## 2. Materials and Methods

### 2.1. Calf Management

This observational study was conducted using the herd health records of a large commercial dairy farm associated with the Michigan State University Training Center for Dairy Professionals (Elsie, MI, USA). This farm has an average of 3500 lactating Holstein cows with a rolling herd average milk production of 12,250 kg/cow and a maternity pen staffed 24 h a day. The newborn care protocol included administering an oral vaccine against diarrhea pathogens (Calf-Guard; Zoetis Inc., Kalamazoo, MI, USA), an intranasal vaccine against respiratory pathogens (Inforce 3; Zoetis Inc., Kalamazoo, MI, USA), and 3 mL of a vitamin E and selenium complex s.c. (MU-SE, Merck Animal Health; Madison, NJ, USA) immediately after birth. Thirty min after administration of vaccines, calves are weighed and subsequently receive 3 L of >22% Brix fresh or frozen colostrum via an orogastric tube. When colostrum stores are available, calves receive another 2 L of >21% Brix colostrum approximately 5–6 h after the first feeding via nipple bottle, but calves are tubed again if needed to ensure ingestion of 2 L of colostrum. Calves not receiving a second meal of colostrum received instead 2 L of milk replacer (27% protein/20% fat in cold months and 28% protein/10% fat in warm months; Cow’s Match, Land O’Lakes Inc., Arden Hills, MN, USA). Farm staff record which calves receive a second colostrum feeding.

Calves are individually housed in stalls in a barn and fed 3 L of milk replacer (Cow’s Match, Land O’Lakes Inc., Arden Hills, MN, USA) 3 times/day until 7 days of age. Subsequently, calves are raised preferentially in groups of up to 22 calves using automatic feeding systems (Calf feeder CF1000S; DeLaval, Kansas City, MO, USA), or in individual stalls and bucket-fed 3 L of milk replacer 3 times/day until weaning. A step-down weaning approach is used in both feeding systems. Water is available ad libitum from birth, and starter concentrate (20% protein, 2.5% fat, 7.5% fiber) from 1 week of age. All heifer calves are managed under identical conditions after weaning in free stall pens grouped according to age and reproductive status. Heifers older than 12 months of age and weighing more than 363 kg are inseminated by trained farm staff based on observed standing estrus, and pregnancy is confirmed via rectal ultrasonography 30 to 40 days after insemination.

### 2.2. Records

At the study farm, a random sample of 12–20 calves aged 2 to 7 days old is sampled every week for serum total protein (STP) determination by the herd veterinarians. Blood samples are collected via jugular venipuncture using 18 G needles and 10 mL vacuumed tubes (Serum Blood Collection Tubes, Becton, Dickinson and Company, Franklin Lakes, NJ, USA), allowed to clot at room temperature, and centrifuged at 2000× *g* for 10 min. Subsequently, serum is collected and analyzed for STP using an optical refractometer (J-351 Clinical Refractometer, Jorgensen Laboratories Inc., Loveland, CO, USA). The refractometer is zeroed with distilled water before measuring each batch of samples. Results are recorded in a database in Access (Microsoft, Redmond, WA, USA). For this study, the STP data collected from January 2014 to April 2017 and used in a previous study were utilized [19]. These years were selected for ensuring that all of the calves had the opportunity to reach the first lactation at the start of data extraction. Calves were classified as experiencing FTPI when STP < 5.2 g/L [20].

Information regarding calf treatment for disease and mortality, growth, reproductive performance, removal from herd, and first lactation 305-d Mature Equivalent milk production (305ME) was extracted from the farm’s software database (DairyComp 305, Valley Agricultural Software, Tulare, CA, USA). Disease diagnosis and treatment were performed by farm personnel following the protocols designed by the herd veterinarians, which are based on the health scoring system developed by McGuirk and Peek [21]. Treatment for diarrhea included oral or intravenous fluid therapy and antimicrobial therapy based on the severity of the disease. Calves diagnosed with bovine respiratory disease (BRD) were treated with gamithromycin 6.6 mg/kg s.c. (Zactran, Boehringer Ingelheim Animal Health USA Inc., Duluth, GA, USA). Average daily gain (ADG) at weaning was calculated based on the recorded weights at birth and weaning ([weaning weight—birth weight]/age (days) at weaning). Female and male calves are managed identically until weaning in the study farm. Thus, both sexes’ data were used to study pre-weaning variables (FTPI, morbidity, and mortality risks). In contrast, only the female calves’ data were used to investigate ADG, reproductive variables, and first lactation 305ME.

### 2.3. Handling of Data

STP records (*n* = 4489) from the period of interest were exported to a spreadsheet (Excel, Microsoft). For each calf with an STP determination, the following information was retrieved from the farm’s software and imputed into the spreadsheet: Sex, birth weight, weaning weight and date, date of disease events, date of removal from the herd, the reason for removal, date of first insemination, number of inseminations, calving date, and first lactation 305ME. Following data entry for all of the above event dates, the calf’s age was calculated at each event. A total of 153 calves were removed from the dataset before analysis because their records were incomplete (*n* = 146) or were assessed by the veterinarian as dehydrated (*n* = 7) when the blood was collected based on skin tenting and degree of eye globe recession. Thus, the records of 2064 male and 2272 female calves were finally analyzed. These records were examined with plots and descriptive statistics to determine their distributions and frequencies in order to identify potential inputting mistakes. Moreover, the records of a randomly selected subset of 140 calves were also checked.

### 2.4. Statistical Analyses

All analyses were performed using the software R [22] and packages “plyr” [23], “survival” [24], “tidyverse” [25], “forestplot” [26], “survminer” [27], and “SurvRegCensCov” [28]. Due to the presence of right-censored data (e.g., mortalities, removal from the herd before the first insemination, failure to become pregnant, pregnancy losses, etc.), survival analysis methods were utilized. Cox-proportional hazard models were used to estimate the association of a second colostrum feeding with pre-weaning morbidity and mortality, age at first insemination, and age at first calving. The following pre-weaning diseases were investigated as grouping variables: BRD, diarrhea, and “any disease pre-weaning” (BRD, diarrhea, or other disease before weaning (e.g., umbilical abscess)). To account for potential confounders, the type of birth (singleton vs. twin), season of birth (fall, winter, spring, and summer), feeding system (automatic vs. bucket feeding), birth weight, and the %Brix of the first colostrum meal were also explored as explanatory variables. Sex (male or female) was also offered to the models when relevant (e.g., only heifers were used for analysis of growth, reproductive, and production variables). Univariable and multivariable analyses were used. Variables with a *p* < 0.20 in the univariable analyses were retained for multivariable analysis. The multivariable models were built using a backward Wald selection method, and explanatory variables with a *p* < 0.05 were considered significantly associated with the outcome and retained in the final models. The proportional hazard assumption in Cox models was verified visually through plots of scaled Schoenfeld residuals against time. The proportional hazard assumption was violated for the variables BRD (Global Schoenfeld test *p* = 0.039) and any disease pre-weaning (*p* = 0.032). Thus, Weibull accelerated failure time regression models were fitted instead for these variables using the same approach. The adequacy of the Weibull distribution was assessed by plotting the log time versus the log of the estimated cumulative hazard estimate. A Kaplan-Meier survivor function was used to estimate the non-disease probability by days of age based on the number of colostrum meals received using the log-rank test to compare groups.

Logistic regression was utilized to investigate the association of a second colostrum feeding with FTPI. A generalized linear model with Poisson distribution was used to investigate the impact of receiving 2 feedings of colostrum on the number of inseminations received by heifers. Linear regression models were used to assess the association between two feedings of colostrum and the continuous variables ADG and first lactation 305ME. Logistic, generalized linear, and linear regression models were constructed as described for the proportional hazard models for the same explanatory variables. Model assumptions were assessed by the evaluation of homoscedasticity and normality of residuals. Normality of residuals was checked by visually assessing histograms and quantile-quantile plots. All reported *p* values are those adjusted for multiple comparisons via Bonferroni correction. Statistical significance was declared at *p* < 0.05.

## 3. Results

Overall, 180 calves received only one colostrum feeding, and 4156 received a second colostrum meal in the first few hours of life (Table 1). The median (interquartile range) of %Brix of the first and second colostrum feedings were 27.6 (4.3) and 25.6 (3.7), respectively, with no differences in the first feeding between calves receiving one or two meals of colostrum (27.1 (5.2) vs. 27.6 (4.7), Wilcoxon test *p* = 0.32). Birth weight was also similar (*p* = 0.20) between feeding groups with a median of 39.5 (4.5) and 40 (7.0) kg in the one and two meal groups, respectively. There were also no differences in median birth weight between female and male calves (39 (5.9) vs. 40.3 (8.2) kg; *p* = 0.10). The median (interquartile range; range) age at weaning was 72 (11; 58–83) d with a median ADG of 0.77 (0.16) kg/d. The majority (90.9%) of the dataset calves were raised in the bucket-fed system during the pre-weaning stage, and calves born from twin pregnancies represented only 1.45% of the total number of calves.

The prevalence of FTPI, BRD, diarrhea, and any pre-weaning disease, separated by sex, season, type of birth, rearing system, and colostrum feeding groups (1 vs. 2 feedings), are reported in Table 2. Calves raised in auto-feeders showed a higher prevalence of BRD, diarrhea, and any pre-weaning disease, but lower pre-weaning mortality than bucket-fed calves. Similarly, calves born from twin pregnancies also showed higher pre-weaning morbidity, but lower mortality than singleton-born calves.

**Figure 1:**
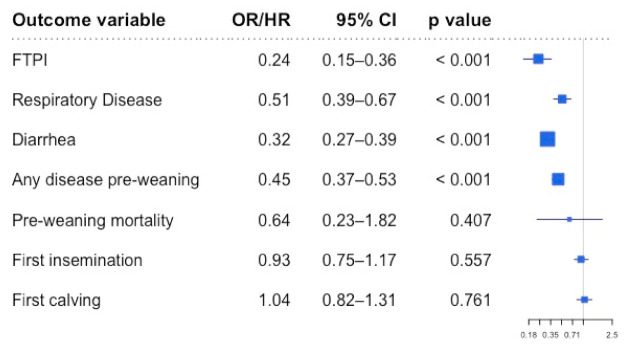
Forest plot showing the association of receiving a second colostrum feeding with failure of transfer of passive immunity (FTPI), pre-weaning disease and mortality, age at first insemination, and age at first calving. Results are reported considering one feeding of colostrum as the reference. The estimate is reported as odds ratio (OR) from logistic regression for FTPI and as Hazard Ratio (HR) from accelerated failure time (respiratory disease, any disease pre-weaning) or Cox proportional hazards (diarrhea, pre-weaning mortality, first insemination, and first calving) analysis.

**Figure 2:**
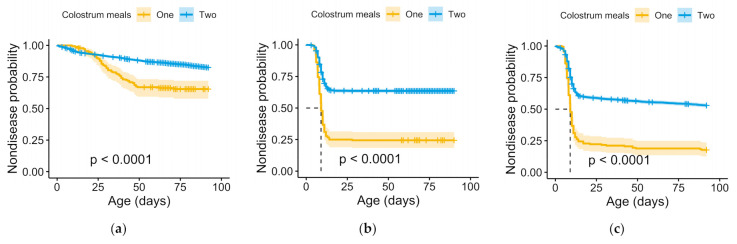
Kaplan-Meier survival curve of the non-disease probability of calves dependent on the number of colostrum meals received within the first 24 h of life for (**a**) respiratory disease, (**b**) diarrhea, and (**c**) any disease pre-weaning. *p* value based on the log-rank test.

The odds of FTPI were four times lower in calves that received a second colostrum feeding than in those that only received one (Figure 1). Calves receiving a second meal of colostrum were also 2 times less likely to be diagnosed with BRD, 3 times less likely to be diagnosed with diarrhea, and 2.3 times less likely to have a history of any disease before weaning (Figure 1). In addition, among the animals that required treatment for diseases pre-weaning, calves that received 2 meals of colostrum were treated at an older age (Table 3, Figure 2). Nevertheless, there were no differences in the hazard of mortality pre-weaning between calves that received two feedings of colostrum and those that only received one (Figure 1).

Calves receiving a second colostrum feeding showed greater ADG at weaning than calves that only received one meal of 3 L of colostrum (Table 4). However, there were no differences in the hazard of first insemination or calving, although there was a trend (*p* = 0.066) for heifers receiving only 1 colostrum meal to get more inseminations than those receiving 2 feedings. Moreover, heifers that received 2 meals of colostrum tended (*p* = 0.081) to produce 984 kg of 305ME more in their first lactation than heifers that only received 1 colostrum meal in their first day of life.

## 4. Discussion

Administration of colostrum within 2 h after birth is recommended to maximize the efficiency of absorption of colostral IgG [12]. However, the calves’ intestine is still permeable to IgG past 12 h and, therefore, a second meal can further increase the transfer of passive immunity [29]. However, Lombard, et al. [13] showed that excellent transfer of passive immunity—defined as serum IgG > 25 g/L—was achieved in several farms feeding a sufficient amount of quality-tested colostrum in one meal soon after birth. Therefore, a second feeding of colostrum may not be needed for transfer of passive immunity when a sufficient mass of immunoglobulins is provided in the initial meal. Our results, however, demonstrated a 4-times reduction in the odds of FTPI in calves receiving a second feeding of 2 L of colostrum compared to those that only received the initial meal of 3 L. Only colostrum with %Brix > 22% was fed to the calves in this study. Thus, the initial feeding provided at least 150 g IgG, the minimum mass of IgG that should be delivered to calves shortly after birth to ensure adequate transfer of passive immunity [30]. Therefore, providing a second colostrum meal 5–6 h after the first postnatal feeding significantly reduces the likelihood of FTPI even in calves receiving adequate IgG in the first meal. In this study, we used the STP threshold of 5.2 g/dL to classify FTPI animals because it was the recommended cut-off to rule in FTPI [20], which was the aim of this study. Thus, using a higher threshold for identifying calves with FTPI in the context of the current study would have led to misclassification bias. Nevertheless, recent recommendations suggest that establishing different classes of STP would be more beneficial than a dichotomic approach [13].

Receiving a second colostrum meal also reduced the probability of being treated for BRD, diarrhea, or any disease before weaning by 48%, 68%, and 56%, respectively. This could be due to the observed lower odds of FTPI, as improved immunity transfer is associated with lower pre-weaning morbidity risk [9,30,31]. Thus, our results support the contention that a second feeding of colostrum is a useful tool to improve pre-weaning calf health.

Moreover, among the calves being treated, the administration of a second colostrum meal was associated with being first treated for diseases later in the pre-weaning period than calves that received only the first meal, suggesting a prolonged protection effect of passive immunity in calves receiving two meals of colostrum. The mean survival age for diarrhea in calves receiving only one colostrum feeding was at two weeks of age, in line with previous studies [31,32,33]. In contrast, the average age at first treatment for diarrhea for those receiving a second colostrum meal was at four weeks of age. Given that the age at which calves experience diarrhea is associated with the enteropathogens involved [34], it is possible that the extra colostrum feeding provided additional protection against the pathogens that cause diarrhea in the first 2 weeks of life, such as enteropathogenic *Escherichia coli*, coronavirus, or rotavirus. Thus, the diarrhea in calves in the second meal group could have been caused by different pathogens than those affecting the calves that only received one meal of colostrum. However, this speculation could not be confirmed in this study because an etiological diagnosis was not sought for diarrhea cases.

There also was a difference in the mean age of BRD treatment between calves fed one colostrum meal (5 weeks) and two colostrum meals (10 weeks). This is likely to have significant influence on the overall impact of BRD in herds because BRD affects heifer growth [35]. Thus, the delayed onset of BRD seen in calves receiving two colostrum feedings might contribute to minimizing the impact of BRD on heifer pre-weaning growth, a factor known to influence heifer health, age at first calving, and lifetime productivity [36,37,38]. Nevertheless, mean survival times are subjected to bias due to the large skew encountered in the distribution of most survival data [39]. As such, the differences in survival time might not be as pronounced as reported. Unfortunately, however, median survival times, which are not subjected to this bias, could not be calculated for all the feeding groups and diseases as less than 50% of the population developed the condition. However, mean survival still supplies valuable information when comparing the performance of 2 groups [40].

Although calves experiencing FTPI are at a higher risk of pre-weaning mortality [9,30,31,41], we did not detect an association between 2 feedings of colostrum and pre-weaning mortality despite observing a reduction in the odds of FTPI in association with a second meal of colostrum. Nevertheless, the overall pre-weaning mortality risk in the dataset was 1.36%, lower than the 5.0% reported by Urie et al. [31], and the 8 to 11% reported in previous US studies [32,33]. Thus, it is possible that the low pre-weaning mortality observed in our study does not allow us to elucidate the potential impact of a second colostrum meal on pre-weaning mortality. This contention is further supported by the broad hazard ratio confidence intervals documented in Figure 1.

In heifers, a second feeding of colostrum was associated with a 130 g/day greater pre-weaning ADG (Table 3). Colostrum is also rich in bioactive compounds such as hormones and growth factors that influence post-natal development, metabolism, and growth [16,17,18]. Thus, it is possible that a second meal of colostrum programmed the calves for better feed efficiency pre-weaning. However, this extent could not be tested in this retrospective study as solid feed intake was not monitored. Moreover, optimized growth during the pre-weaning stage is critical for greater reproductive efficiency and lifetime production [38,42,43]. However, we did not observe differences in the age at first insemination or calving between heifer calves that received one or two meals of colostrum, and only a tendency for heifers receiving two meals to require fewer inseminations and have a higher first lactation 305ME.

The administration of a second meal of colostrum was associated with a reduced hazard of disease pre-weaning, and calves experiencing pre-weaning disease experience decreased reproductive efficiency later in life [36]. Indeed, our previous study documented a 5–7 day delay in age at first insemination and calving along with reduced hazards of insemination and calving in heifers experiencing pre-weaning disease in the same dataset [19]. However, despite the observed positive effect of 2 meals of colostrum on pre-weaning disease, we found no relationship between the number of colostrum feedings and pubertal or calving age. This could be attributed to the intensive feeding program followed in the study farm. The majority (73.9%) of heifers in the dataset had an ADG > 0.7 kg/day at weaning [19], the rate required to reach growth targets for first calving at 24 months of age, which, in turn, is associated with increased productivity and longevity [44]. Thus, the feeding regime at the farm may have allowed heifers to meet adequate pre-weaning growth rates irrespective of the number of colostrum meals received postnatally. Hence, the impact of a second meal of colostrum on heifers raised under more restrictive liquid feeding approaches needs to be evaluated further.

This study was conducted on just one farm. Although this allows for more homogeneous diagnosis, treatment, and management practices, there might be farm-specific factors that influence the results observed. In addition, the disease status of the animals was determined by farm staff using a standardized method based on health scores. However, this method is known to have only moderate sensitivity (55.4%) and specificity (58.0%) for identifying calves with lung lesions [45]. Hence, the accuracy of the diagnostic method might have influenced the results, as some calves could have been misclassified as diseased or healthy. Furthermore, the study’s retrospective observational nature precludes us from making cause-effect relationships, as calves were not randomly assigned to groups and received only one feeding of colostrum in periods of shortages of stores, resulting in imbalanced groups sizes and associated differences in variance, which could have influenced the results of the statistical analyses. Similarly, there was a slightly higher number of calves in the one meal group during fall and winter, when it has been documented that cows produce lower volumes of colostrum [46]. Hence, it is possible that seasonality could have affected the results observed in this study. Nevertheless, we accounted for the season in which calves were born in our statistical analyses. Furthermore, it is also possible that factors affecting colostrum production may also affect calf health and performance, as maternal late-gestation status is known to influence offspring’s growth, productivity, metabolism, and health [47,48]. Thus, it cannot be excluded that calves born during periods of colostrum shortage were also subject to negative in utero effects on postnatal health, growth, and reproductive and production performance. To our knowledge, however, this is the first study comparing the health and productivity of calves receiving a second meal of colostrum in addition to an adequate amount of quality-tested colostrum soon after birth. Multi-herd randomized controlled trials are now needed to confirm the positive impact of a second feeding of colostrum in dairy calf health observed in our study.

## 5. Conclusions

Based on the associations observed, our results suggest that feeding calves a second feeding of 2 L of good-quality colostrum 5 to 6 h after an initial feeding of 3 L of quality-tested colostrum soon after birth could be an effective strategy to decrease FTPI and morbidity and optimize ADG in dairy calves pre-weaning even when calves received sufficient colostrum in the first meal. Nevertheless, further randomized controlled studies are needed to establish cause-effect relationships.

## Figures and Tables

**Table 1:** Distribution of the calves enrolled based on whether they received one or two meals of colostrum in the first hours of life.

	Colostrum Meals	
	Two	One
Sex		
Male	2020 (48.6%)	44 (24.4%)
Female	2136 (51.4%)	136 (75.6%)
Year of birth		
2014	1028 (24.7%)	41 (22.8%)
2015	1402 (33.7%)	57 (31.7%)
2016	1315 (31.6%)	68 (37.8%)
2017 (until April)	441 (10.6%)	14 (7.8%)
Season of birth		
Fall	1214 (29.2%)	49 (27.2%)
Winter	960 (23.1%)	54 (30.0%)
Spring	949 (22.8%)	41 (22.8%)
Summer	1033 (24.9%)	36 (20.0%)
Type of birth		
Singleton	4096 (98.6%)	177 (98.3%)
Twin	60 (1.4%)	3 (1.7%)
Rearing system		
Auto-fed	372 (8.9%)	23 (12.7%)
Bucket-fed	3784 (91.1%)	157 (87.2%)

Results expressed as the number of calves in each group and the corresponding percentage within the meal group.

**Table 2:** Descriptive statistics of the prevalence of failure of transfer of passive immunity (FTPI), causes of pre-weaning morbidity, and pre-weaning mortality based on the sex, season, and type of birth, rearing system, and number of colostrum meals of the calves analyzed in the study.

	*n*	FTPI Prev.^1^	Respiratory Disease		Diarrhea		Any Disease		Mortality	
			Prev. ^1^	Age (Days) at Diagnosis ^2^	Prev. ^1^	Age (Days) at Diagnosis ^2^	Prev. ^1^	Age (Days) at Diagnosis ^2^	Prev. ^1^	Age (Days) at Death ^3^
Sex										
Female	2272	9.86%	21.40%	30 (40)	40.80%	9 (3)	53.60%	9 (5)	1.19%	13 (28)
Male	2064	10.00%	13.20%	27 (43)	35.00%	9 (3)	42.60%	9 (4)	1.55%	20 (42)
Season of birth										
Fall	1263	8.70%	17.40%	38.5 (47)	38.20%	9 (2)	49.70%	9 (4)	0.95%	14.5 (44)
Winter	1014	7.80%	22.60%	31 (27)	38.10%	9 (4)	50.00%	10 (4)	2.37%	19.5 (57)
Spring	990	12.70%	15.70%	24 (41)	37.20%	9 (3)	46.40%	9 (5)	1.21%	15.5 (22.5)
Summer	1069	11.00%	14.70%	18.5 (52)	38.50%	9 (3)	47.10%	9 (4)	1.03%	17 (24)
Type of birth										
Singleton	4273	10.00%	17.40%	30 (41)	37.80%	9 (3)	48.20%	9 (5)	1.38%	17 (32)
Twin	63	6.40%	26.90%	71 (69)	47.60%	8.5 (3.25)	60.30%	9 (4.25)	0%	–
Rearing system										
Auto-fed	395	11.80%	41.20%	24 (21)	47.30%	10 (4)	72.40%	11 (8)	0.51%	13.5 (1)
Bucket-fed	3941	9.80%	15.10%	34 (48.5)	37.10%	9 (3)	46.00%	9 (4)	1.45%	17 (33)
Number of colostrum meals										
Two	4156	9.40%	16.80%	30 (44)	36.40%	9 (4)	46.90%	9 (5)	1.32%	17 (32)
One	180	22.2%	33.9%	30 (16)	75.6%	8 (3)	82.2%	9 (3)	2.22%	38 (54)
Overall	4336	9.9%	17.6%	30 (41)	38.0%	9 (3)	48.4%	9 (5)	1.36%	17 (32)

^1^ Prev. = Prevalence. ^2^ Median (interquartile range) age at first disease diagnosis. ^3^ Median (interquartile range) age at death.

**Table 3:** Survival time (age in days) at disease diagnosis pre-weaning based on the number of colostrum meals.

Disease Group	Colostrum Meals						*p* Value ^1^
	Two			One			
	Median	Mean	95% CI	Median	Mean	95% CI	
Respiratory disease	– ^2^	80.6	79.5–81.7	– ^2^	56.7	53.4–60.0	<0.001
Diarrhea	– ^2^	34.4	33.5–35.2	9	12.8	11.5–14.1	<0.001
Any pre-weaning disease	– ^2^	53.3	51.6–55.0	9	21.7	17.1–26.3	<0.001

^1^*p*-value based on the log-rank test for survival. ^2^ A median time could not be calculated because less than 50% of the population developed the condition.

**Table 4:** Comparison of pre-weaning average daily gain, number of inseminations, and 305ME between heifers receiving 1 or 2 colostrum meals after birth. Results reported as estimated means and 95% CI.

	Number of Colostrum Meals		*p* Value
	Two (*n* = 2126)	One (*n* = 136)	
Average daily gain (kg/d)	0.86 (0.79–0.93)	0.74 (0.64–0.84)	<0.001
Number of inseminations	1.84 (1.78–1.90)	2.13 (1.88–2.38)	0.066
First lactation 305ME (kg)	16,424 (15,249–18,329)	15,440 (15,067–18,693)	0.081

## Data Availability

The data that support the findings and which are presented in this study are available on reasonable request from the corresponding author, Angel Abuelo, abuelo@msu.edu. The data is not publicly available as not all data of the study has been published yet.
